# Azithromycin-Resistant *Salmonella enterica* Serovar Typhi AcrB-R717Q/L, Singapore

**DOI:** 10.3201/eid2702.203874

**Published:** 2021-02

**Authors:** Sophie Octavia, Ka Lip Chew, Raymond T. P. Lin, Jeanette W. P. Teo

**Affiliations:** National Centre for Infectious Diseases, Singapore (S. Octavia, R.T. P. Lin);; National University Hospital, Singapore (K.L. Chew, R.T. P. Lin, J.W.P. Teo)

**Keywords:** Salmonella enterica, bacteria, serovar Typhi, AcrB-R717Q/L, azithromycin, azithromycin-resistant isolates, antimicrobial resistance, typhoid fever, Singapore

## Abstract

Global travel has led to intermittent importation of multidrug-resistant *Salmonella* *enterica* serovar Typhi into industrialized countries. We detected azithromycin-resistant *Salmonella* Typhi in Singapore, of which 2 isolates were likely locally acquired. Ongoing vigilance and surveillance to minimize the public health risk for this serious pathogen is needed.

In Singapore, the incidence of typhoid fever is low (0.8–1.2 cases/100,000 population annually). Most cases are imported, particularly from the South Asia subcontinent (*1*). First-line treatments include ampicillin, trimethoprim/sulfamethoxazole, and chloramphenicol. With increasing multidrug-resistant and fluoroquinolone-resistant infections, ceftriaxone and azithromycin are the next treatment alternatives. However, resistance to ceftriaxone or azithromycin resistance has been reported (*2*).

Multidrug-resistant *Salmonella* Typhi isolates belong to haplotype H58 (genotype 4.3.1), which is predominant in Asia and Africa (*3*). Resistance in genotype 4.3.1 is characterized by nonsynonymous mutations in the quinolone resistance–determining-region (QRDR) of DNA gyrase genes *gyrA* and *gyrB*, DNA topoisomerase IV genes *parC* and *parE*, and acquisitions of IncHI1 plasmids (*3*,*4*). Azithromycin-resistant *Salmonella* Typhi is also seen in this genotype (*2*).

During September 2019–April 2020, increased MICs for azithromycin were detected for 3 *Salmonella* Typhi isolates identified at the National University Hospital, Singapore. To characterize the molecular mechanisms of azithromycin resistance and genetic lineage in these isolates, we performed whole-genome-sequencing.

## The Study

This study was approved by the National Healthcare Group Domain Specific Review Board (study no. 2020/01010). Apart from the 3 isolates tested for azithromycin resistance, an additional 21 *Salmonella* Typhi isolates (total 24 isolates) collected during 2016 and 2020 at the National University Hospital, a 1,200-bed tertiary hospital, were retrospectively investigated.

Genus was identified by using the Bruker MALDI Biotyper (Bruker Daltonics, https://www.bruker.com), and serotyping was performed by using slide agglutination and antiserum (Statens Serum Institute, Copenhagen, Denmark). After genus and serotype were confirmed, these isolates were submitted to the National Public Health Laboratory, Singapore, as part of the national surveillance program for *Salmonella* spp.

Drug susceptibility testing was routinely performed by using Vitek 2 (bioMérieux, https://www.biomerieux.com) and supplemented by using the Etest (bioMérieux) for ciprofloxacin and azithromycin. Azithromycin MICs were further confirmed by using broth microdilution. Quality control isolates used were *Escherichia coli* ATCC 25922, *Salmonella* Enteritidis ATCC 13076, *Salmonella* Typhimurium ATCC 14028, and *Staphylococcus aureus* ATCC 29213. EUCAST interpretative breakpoints were used, including for azithromycin resistance, which is based on the tentative epidemiologic cutoff value (https://eucast.org/fileadmin/src/media/PDFs/EUCAST_files/Breakpoint_tables/v_10.0_Breakpoint_Tables.pdf).

Whole-genome sequencing was performed by using MiSeq (Illumina, https://www.illumina.com) to generate 300-bp paired-end reads. Raw reads were assembled by using Shovill (https://github.com/tseemann/shovill). Isolates were genotyped by using the GenoTyphi tool (https://github.com/katholt/genotyphi), which separates *Salmonella* Typhi isolates into clades on the basis of the extended genotyping framework described by Wong et al. (*4*). SRST2 (*5*) was used to determine the presence of acquired antimicrobial drug resistance genes by using the ResFinder database (*6*). Chromosomal QRDR mutations in *gyrA*, *gyrB*, and *parC*, as well as the efflux pump AcrB (*acrB*-R717Q) mutations conferring resistance to azithromycin, were also investigated by using the GenoTyphi tool. PlasmidFinder (https://cge.cbs.dtu.dk/services/PlasmidFinder/) was used to detect replicons. 

*Salmonella* Typhi CT18 (GenBank accession no. AL513382.1) was designated as the reference genome. We also downloaded all publicly available *Salmonella* Typhi genome sequences belonging to lineage 4.3.1 and its sublineages from the Pathogenwatch database (https://pathogen.watch) for comparison with our isolates belonging to lineage 4.3.1. Core-genome single-nucleotide polymorphisms were obtained by using snippy pipeline (https://github.com/tseemann/snippy) and then used to generate a phylogenetic tree by using FastTree (*7*). The resulting tree was visualized by using iTOL version 4 (*8*). Raw reads have been submitted to the Sequence Reads Archive under BioProject no. PRJNA660881.

Whole-genome sequencing results showed that 15 of the 24 *Salmonella* Typhi isolates belonged to subclade 4.3.1 (haplotype H58), which can be further differentiated into 4.3.1.1 (4/15), 4.3.1.2 (8/15), and 4.3.1.3 (3/15) (Table). The 4.3.1 subclade is a dominant lineage disseminating from South Asia into East Africa (*3*). Signature mutations associated with this subclade are QRDR mutations at codon positions 83 and 87 in *gyrA* conferring fluoroquinolone resistance. The phylogenetic tree showed that these isolates did not form a unique group but were interspersed with isolates from countries in South Asia, particularly Bangladesh (Figure). The remaining 9/24 isolates belonged to subclades 0.0.2 (n = 1), 2.3.3 (n = 4), 3 (n = 2), 3.2.1 (n = 1), and 4.1 (n = 1).

**Table:** Characteristics of 15 *Salmonella enterica* serovar Typhi subclade 4.3.1 isolates tested for azithromycin resistance, Singapore*

Genotype and no.	Isolate name	Culture source	Patient travel history	Chromosomal azithromycin mutation	QRDR mutations	Other acquired resistance determinants	Plasmid replicon	Drug and MIC, mg/L				
								Azithromycin BMD	Ampicillin	Cefotaxime	Ciprofloxacin	Trimethoprim/sulfamethoxazole
4.3.1.1, n = 4	SLT0596	Unknown	Pakistan	–	GyrA-S83F	*bla*_TEM-1_,*catA1,dfrA7,sul1,sul2*	IncFIB	2 (S)	≥32.0 (R)	≤1.0 (S)	0.38 (R)	≥320.0 (R)
	SLT10	Blood	Bangladesh	–	GyrA-S83F	*bla*_TEM-1_,*catA1,dfrA7,sul1,sul2*	IncFIB	4 (S)	≥32.0 (R)	≤1.0 (S)	0.25 (R)	≥320.0 (R)
	SLT0291	Blood	None recent	AcrB-R717Q	GyrA-S83F	*bla*_TEM-1_,*catA1,dfrA7,sul1,sul2*	IncFIB	32 (R)	≥32.0 (R)	≤1.0 (S)	0.38 (R)	≥320.0 (R)
	SLT0892	Blood	Bangladesh	AcrB-R717Q	GyrA-S83F	*catA1,dfrA7,sul1*	–	32 (R)	≤2.0 (S)	≤1.0 (S)	0.38 (R)	≥320.0 (R)
4.3.1.2, n = 8	SLT3	Stool	Myanmar	–	GyrA-D87N,GyrA-S83F,ParC-S80I	–	–	4 (S)	≤2.0 (S)	≤1.0 (S)	>32 (R)	≤20.0 (S)
	SLT0096	Stool	India	–	GyrA-D87N,GyrA-S83F,ParC-S80I	–	–	8 (S)	≤2.0 (S)	≤1.0 (R)	0.38 (R)	≤20.0 (S)
	SLT2	Stool	Philippines	–	GyrA-D87N,GyrA-S83F,ParC-S80I	–	–	4 (S)	≤2.0 (S)	≤1.0 (S)	>32 (R)	≤20.0 (S)
	SLT1131	Blood	India and United States	–	GyrA-D87N,GyrA-S83F,ParC-S80I	–	–	4 (S)	≤2.0 (S)	≤1.0 (S)	8 (R)	≤20.0 (S)
	SLT4	Blood	None recent	–	GyrA-S83F	–	–	4 (S)	≤2.0 (S)	≤1.0 (S)	0.5 (R)	≤20.0 (S)
	SLT0469	Stool	India	–	GyrA-S83F	–	–	4 (S)	≤2.0 (S)	≤1.0 (S)	0.5 (R)	≤20.0 (S)
	SLT0626	Blood	India	–	GyrA-S83F	–	–	8 (S)	≤2.0 (S)	≤1.0 (S)	0.38 (R)	≤20.0 (S)
	SLT11	Blood	India	–	GyrA-S83F	–	–	4 (S)	≤2.0 (S)	≤1.0 (S)	0.38 (R)	≤20.0 (S)
4.3.1.3, n = 3	SLT8	Blood	Bangladesh	–	GyrA-S83F	*bla*_TEM-1_,*qnrS,sul2,tet(A)*	IncFIB(K)	4 (S)	≤2.0 (S)	≤1.0 (S)	0.25 (R)	≤20.0 (S)
	SLT1	Blood	Bangladesh	–	GyrA-S83F	*bla*_TEM-1_,*qnrS,sul2,tet(A)*	IncFIB(K)	4 (S)	≥32.0 (R)	≤1.0 (S)	4 (R)	≤20.0 (S)
	SLT1105	Blood	None recent	AcrB-R717L	GyrA-S83F	*bla*_TEM-1,_ *qnrS,sul2,tet(A)*	IncFIB(K)	16 (S)	≥32.0 (R)	≤1.0 (S)	4 (R)	≤20.0 (S)

*BMD, broth microdilution, QRDR, quinolone resistance–determining-region; R, resistant; S, susceptible; –, not detected,

**Figure:**
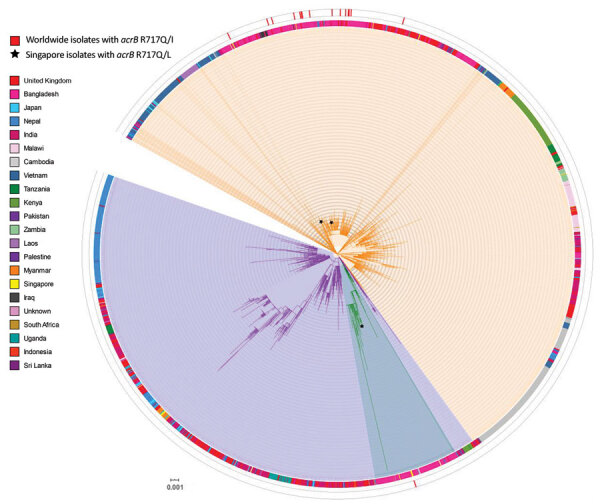
Core single-nucleotide polymorphism phylogenetic tree of 15 genotype 4.3.1 *Salmonella enterica* serovar Typhi isolates tested for azithromycin resistance, Singapore. Isolates sequenced in this study were compared with other publicly available *Salmonella* Typhi genomes, indicated by their corresponding GenBank accession number obtained from Pathogenwatch (https://pathogen.watch) on the basis of 3,104 core-genome single-nucleotide polymorphisms. Azithromycin-resistant isolates analyzed in this study are indicated by asterisks (*). *Salmonella* Typhi CT18 was designated as the reference genome (blue). Genotype information obtained from the GenoTyphi tool (*4*) was included for all genomes, and country of isolation was added when available. The tree was illustrated by using iTOL version (*8*). Scale bar indicates nucleotide substitutions per site.

The genomic antimicrobial drug–susceptibility profiles correlated with the phenotypic susceptibilities (Table). Six isolates harbored *bla*_TEM-1_, but none had extended-spectrum-β-lactamases or carbapenemases. All isolates were resistant to ciprofloxacin and had QRDR mutations (Table). Four of 8 isolates belonged to subclade 4.3.1.2 and had the triple QRDR mutation combination. Only isolates with *dfrA7*, *sul1*, and *sul2* were phenotypically resistant to trimethoprim/sulfamethoxazole.

The 3 azithromycin-resistant isolates have not acquired macrolide-modifying enzymes, such as methylases [*erm(A)*, *erm(B)*, and *erm(C)*], esterases [*ere(A)* and *ere(B)*], or phosphotransferases [*mph(A), mph(B)*, and *mph(D)*] observed in isolates belonging to the order Enterobacterales (*9*). There were no chromosomal alterations in the 50S ribosomal subunit proteins L4 (*rlpD*) and L22 (*rlpV*) (*11*). Instead, R717Q/L mutations in the efflux pump AcrB were detected. Increased MICs for azithromycin (R717Q: 32 mg/L, R717L: 16 mg/L) were observed for these isolates. Azithromycin MICs <4 mg/L were observed for all wild-type *acrB* isolates (Table). The AcrB-R717Q mutation was reported in azithromycin-resistant *Salmonella* Typhi 4.3.1.1 in Bangladesh (*2*) and subsequently in a Pakistan-specific 4.3.1.1 cluster (*10*). The mutation that emerged in Pakistan is believed to be a de novo spontaneous mutation, rather than spread of an azithromycin-resistant clone (*10*). AcrB-R717Q–associated azithromycin resistance has also been reported in India (*11*).

## Conclusions

The AcrB-R717L mutation (isolate SLT1105) is novel in *Salmonella* Typhi. This mutation was described in an azithromycin-resistant *Salmonella* Paratyphi A isolate in Bangladesh (*2*). Functional analysis of the R717L mutation conferred resistance to a sensitive *Salmonella* Paratyphi A strain resulted in a 4-fold increase in the MIC (7 mg/L vs. 28 mg/L; p = 0.0001) (*2*). In *Salmonella* Typhi, the mutation also appears to impart azithromycin resistance (Table).

These AcrB-R717Q/L mutations were in multidrug-resistant isolates. This finding is worrisome because of the unavailability of oral antimicrobial drug treatment options and increased relapses when treated with β-lactams without intracellular-acting antimicrobial drugs.

Most case-patients had relevant travel history within 2 months before onset of symptoms, including travel to India (n = 5), Bangladesh (n = 4), Pakistan (n = 1), Myanmar (n = 1), and the Philippines (n = 1). Three cases appeared to be local transmission, of which 2 had AcrB-R717Q/L mutations. The remaining case-patient, whose isolate had the AcrB-R717Q mutation, had traveled to Bangladesh and probably acquired the infection in this country (*2*).

Hooda et al. (*12*) analyzed 49,115 *Salmonella* genomes and found the AcrB-R717Q/L mutation in 16 *Salmonella* Typhi genomes (≈0.03%). Although this number was small, the rate of acquisition of such novel mechanisms might hasten, especially with increasing use of azithromycin, such as in mass drug administration with azithromycin as a key component for the control of neglected tropical diseases (*12*). The proportion of isolates in our study with AcrB-717Q/L mutation (20%, 3/15) was unexpectedly higher than previously reported. The reasons for this finding are unclear. However, our study was a single-center study that had a limited number of cases.

Genotypic testing of the usual azithromycin resistance–associated genes in the order Enterobacterales cannot identify *acrB* mutations, and there are currently no formal breakpoints to guide phenotypic testing. An azithromycin MIC of 16 mg/L was observed for 1 isolate with the AcrB-R717L mutation. Although this azithromycin MIC was higher (<8 mg/L) than that for isolates without the mutation, this isolate is still considered wild-type. Detection of increased MICs raises suspicion for resistance requiring further confirmation. Additional data are required to correlate resistance mutations, MICs, and treatment outcomes.
